# Personal and Media Factors Related to Citizens’ Pro-environmental Behavioral Intention against Haze in China: A Moderating Analysis of TPB

**DOI:** 10.3390/ijerph17072314

**Published:** 2020-03-30

**Authors:** Xiaodong Yang, Liang Chen, Lai Wei, Qi Su

**Affiliations:** 1 School of Journalism and Communication, Shandong University, Jinan 250100, China; xyang012@e.ntu.edu.sg (X.Y.); weilai0207@mail.sdu.edu.cn (L.W.); suqi77@mail.sdu.edu.cn (Q.S.); 2 School of Communication and Design, Sun Yat-sen University, Guangzhou 510006, China

**Keywords:** theory of planned behavior, pro-environmental behavior, efficacy messages, threat messages

## Abstract

This study extends the theory of planned behavior by taking communication factors into account to examine the determinants of pro-environmental behavioral intention in haze mitigation. Unlike other theory of planned behavior (TPB) extension studies, we shift the focus of inquiry from examining the mediating role of TPB variables to investigating the moderating role in promoting pro-environmental behavior. Using an online survey in China, the results indicated that attitude, subjective norms, perceived behavioral control, and attention to haze-related efficacy messages were positively associated with pro-environmental behavioral intention. Notably, attention to haze-related threat messages had no significant relationship with behavioral intention. Moreover, moderation analyses revealed that the interaction effects between attention to efficacy messages and attention to threat messages on behavioral intention vary among people with different attitude, subjective norms, and perceived behavioral control. Theoretically, this study contributes to the literature of the TPB by analyzing its moderating role in promoting behavior change. Findings from this study suggest the importance of disseminating distinctive media messages to audiences with different personality traits, which is beneficial for practitioners to tailor specific messages in environmental campaigns.

## 1. Introduction

With the rapid industrialization and urbanization in recent years, haze pollution is becoming a serious environmental issue in China. The latest annual report of environmental status showed that the air quality in over 64% of Chinese urban cities has exceeded the limitation [1]. As one of the most disastrous weather events in China, haze pollution has triggered public anxiety [2]. Despite an urgent need for green behavior change, the citizens’ intention to participate in haze mitigation is low [3]. Thus, it is imperative that a full understanding is gained of the determinant of pro-environmental behavior change.

The theory of planned behavior (TPB) is one of the most prominent theories in examining the determinants of behavior. It posits that individuals’ behavior is shaped by their attitude, social norm, and perceived behavior control [4]. A significant number of studies have demonstrated the role of these factors in shaping pro-environmental behavioral intention [5,6]. Despite these encouraging findings, a number of studies noted that when media consumption is measured, the amount of attention that people pay to media messages could predict future behavior [7,8]. For example, Ho, Liao, and Rosenthal [8] found that both media attention and TPB variables had significant relationships with individuals’ pro-environmental behavioral intention. Thus, a significant number of studies extend the TPB by taking communication variables into account [9,10].

Unlike other environmental issues that focus on something other than personal safety, haze pollution is perceived as a personal risk that exerts direct influence to personal health. As people are motivated to seek information to eliminate uncertainty about risks, the information embedded in media messages becomes a major focus of their attention. Besides, many empirical studies have documented that exposure to messages with different content could lead to different effects on behavior [11,12]. Therefore, this study proposes to examine communication variables in extended TPB by operationalizing media attention in terms of attention to different media content.

In terms of the effects of media content, prior studies on environmental campaigns reported that attention to either efficacy or threat messages had different effects in promoting individuals’ pro-environmental behavioral intention [12,13]. The varied effects of efficacy and threat messages have been extensively discussed in literature on the extended parallel process model (EPPM). This model bases its predictions of behavioral responses to information on people’s appraisals of two key constructs: threat and efficacy [14]. Exposure to messages containing either efficacy or threat information could lead to different behavioral outcomes. Thus, this study proposes to examine the relationships between attention to different media messages and behavioral outcomes, with a particular focus on efficacy and threat messages about haze mitigation.

Moreover, media effects literature underscores audiences’ attributes, as people react differently even if they pay attention to the same messages [15,16]. This implies that audiences’ attributes might be a set of potential moderators in the relationship between media attention and behavioral intention. Correspondingly, empirical research revealed that the combination of threat and efficacy messages could be a motivator as well as an inhibitor for different groups of people [17,18]. These findings suggest a necessity of making an in-depth examination of how the relationships between media attention and behavioral intention vary among different groups of people. Specifically, this study expects that people with different attitude, social norm, or perceived behavioral control would react differently toward media messages about haze mitigation.

The aim of this study is to address the following research gaps in our understanding of pro-environmental behavior promotion. First, little is known about the effectiveness of different media messages in encouraging individuals’ behavioral intention to fight against haze. Most prior studies treat environmental messages as a composite variable, while the effects of different messages in promoting pro-environmental behavior have been largely overlooked [19,20]. Second, how people with different personality traits react to different environmental messages remains largely uninvestigated. Finally, despite extensive research working on TPB extension, there are gaps in our understanding of the moderating effects of the TPB variables in promoting behavior change, since most TPB extension studies focused on the mediating role of the TPB variables in the relationships between communication factors and pro-environmental behavior [9,10].

Driven by these research gaps, this study addresses the following research questions. First, how do the communication factors affect individuals’ pro-environmental behavioral intention to engage in haze mitigation? Second, how do TPB factors—attitude, social norm, and perceived behavior control—affect the behavioral intention? Finally, whether the effects of communication factors on pro-environmental behavioral intention to engage in haze mitigation vary among people with different positive attitude, social norms, and perceived behavior control. For instance, will the effects of communication factors in promoting pro-environmental behavior be stronger for people with more perceived social norms than for those with less perceived social norms?

**Figure 1:**
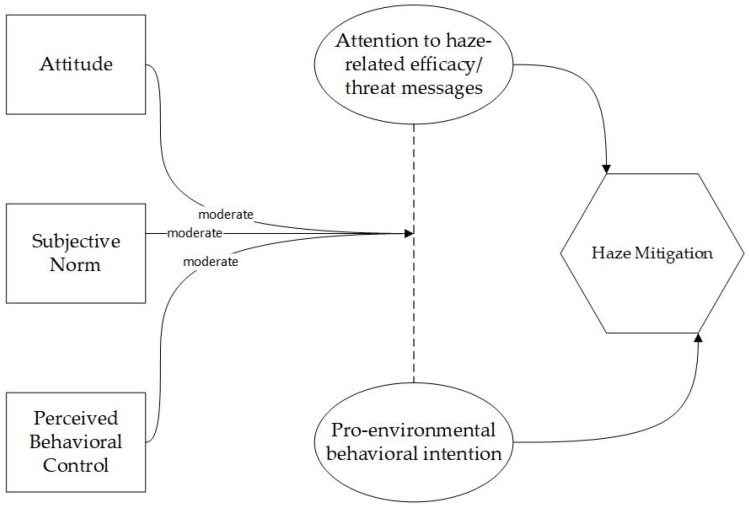
Conceptual model.

Specifically, this study applies the TPB and EPPM, which consider how attitude, social norm, perceived behavioral control, as well as attention to haze-related efficacy messages and threat messages, influence pro-environmental behavioral intention of mitigating haze pollution. In particular, this study conducts a set of moderation analyses to examine how the TPB variables moderate the relationships between attention to haze-related efficacy/threat messages and behavioral intention. Figure 1 shows the research framework of this study.

Findings from this study contribute to the theoretical literature of the TPB by examining its moderating role rather than a mediating role. Instead of investigating the sequential path from communication factors to behavior change via TPB variables, this study explores whether the effects of communication factors on behavior vary among people with different levels of attitude, social norm, or perceived behavior control, which offers a subtle understanding of the moderating effects of the TPB in promoting behavior change. Practically, our findings offer suggestions for practitioners on messages tailoring in environmental campaigns to increase public engagement with haze mitigation. More detailed theoretical and practical implications are given later in the discussion.

## 2. Literature Review and Research Hypotheses

### 2.1. Pro-Environmental Behavioral Intention

A significant body of literature has investigated the barriers to public engagement in pro-environmental behaviors. Numerous theoretical frameworks have been developed to identify the roots of direct and indirect environmental action. As the oldest and simplest models of pro-environmental behavior, the linear model emphasizes that environmental knowledge leading to environmental awareness and concern, which in turn leads to pro-environmental behavior [21]. The rationale underlining this model assumes that providing people with information about environmental issues results in pro-environmental behavior. Numerous studies have empirically demonstrated that media is an important source of information on environmental issues [22,23,24]. Thus, communication factors, such as attention to efficacy and threat messages should be taken into account in the examination of individuals’ pro-environmental behavioral intention to engage in haze mitigation.

Moreover, the theory of planned behavior, one of the most influential models explaining behavior change, has been extensively utilized in the context of environmental issues. By examining personal factors, such as attitude toward certain behavioral, social norms, and perceived behavioral control, many studies have demonstrated their applicability in predicting pro-envionmental behavioral intention [25,26]. Thus, this study proposes to examine the role of TPB constructs as personal factors in promoting pro-environmental behavioral intention in the context of haze mitigation.

### 2.2. Personal Factors

Ajzen [4] developed the TPB to understand the psychological determinants of behavioral change. The TPB proposes three antecedents of behavioral intention: attitude, subjective norm, and perceived behavioral control. The applications of the TPB to explain behavioral intention are widespread in the literature [27,28].

***Attitude.*** In terms of the determinants of behavioral change, attitude toward behaviors has long been recognized as a powerful factor in shaping one’s behaviors. Attitude refers to the degree to which one has a positive or negative appraisal of performing a certain behavior [4]. In general, a favorable attitude with respect to a certain behavior should enhance the intention of an individual to engage in this behavior. This assumption has been supported by empirical evidence [29,30,31]. Moreover, a significant body of research has demonstrated that attitude toward pro-environmental behavior is a strong predictor of individuals’ pro-environmental behavioral intention [32,33,34].

***Subjective Norm.*** Beyond personal attitude, social influence also shapes people’s behaviors. In particular, out of fear of social rejection, people’s normative beliefs motivate them to take action [35]. Since people often engage in social comparisons with their referent groups, they are likely to be influenced by these groups’ beliefs [36]. Subjective norm highlights the perceived social influence toward performing a given behavior. It refers to individuals’ beliefs on the degree to which how important others would expect them to perform a behavior [4]. Thus, people tend to perform the behaviors that are consistent with the subjective norms, which reflect the social expectations that others have toward an individual.

***Perceived Behavior Control.*** Behavioral intention is not only influenced by individuals’ attitude and social norms, but is shaped by the evaluation of their ability to perform specific action. Similar to self-efficacy proposed by Bandura [37] in social cognition theory, perceived behavioral control is individuals’ judgment of their ability to perform the behavior in question [4]. In research on environmental behavior, Bandura [38] pointed out that self-efficacy is an effective indicator for predicting pro-environmental behavior. When individuals believe that they are capable of participating in pro-environmental behavior and can control their behaviors that destroy the environment, their intentions to perform the behavior will be high.

Given a significant number of empirical evidence supports the usefulness of TPB in explaining individuals’ pro-environmental behavioral intention [20,25], we posit the following hypotheses regarding the relationships between TPB variables and behavioral intention to engage in haze mitigation:

H1: Attitude toward pro-environmental behaviors is positively associated with pro-environmental behavioral intention to engage in haze mitigation.

H2: Subjective norm is positively associated with pro-environmental behavioral intention to engage in haze mitigation.

H3: Perceived behavioral control is positively associated with pro-environmental behavioral intention to engage in haze mitigation.

### 2.3. Media Factors

In addition to the factors in TPB, media also play important roles in shaping public’s pro-environmental behavioral intention. Though many studies extended TPB by including media attention, most of them examined how attention across different media platforms accounted for behavioral intention. The effects of messages content remain largely unexamined in TPB extension studies. Nonetheless, several theoretical models on message content and behavioral outcomes, such as the EPPM [14], propose that people react differently to efficacy and threat messages. Therefore, this study extends the TPB by operationalizing media attention in terms of attention to haze-related efficacy and threat messages.

***Attention to Efficacy Messages.*** The haze-related efficacy messages include media content seeking to guide the public to adopt pro-environmental behaviors to alleviate haze pollution. We posit that people who attend more to these messages are more likely to elaborate on and acquire knowledge from the content. According to model of knowledge–attitude–behavior (KAB), by providing information about pro-environmental knowledge, media can change individuals’ environmental attitude and pro-environmental behavior [39]. The information deficit model also points out that the lack of knowledge is a barrier for behavioral change [21]. In addition, numerous studies have demonstrated the effectiveness of informational messages in promoting adoption of pro-environmental behaviors [8,40]. Accordingly, we propose that attention to haze-related efficacy messages can yield similar media effects on pro-environmental behaviors. Hence, the hypothesis is postulated as follows:

H4: Attention to haze-related efficacy messages is positively associated with pro-environmental behavioral intention to engage in haze mitigation.

***Attention to Threat Messages.*** Threatening messages, in the social psychology literature referred to as fear appeals, are widely used in persuasive campaigns. By arousing fear, threat messages motivate people to adopt self-protection behaviors [41]. The haze-related threat messages include media content describing the negative effects caused by haze. These messages can be particularly relevant to environmental behaviors because the public would learn about the severity of and susceptibility to haze pollution from them. Threat messages have been extensively discussed in persuasive theories, such as protection motivation theory, which posits that individuals’ perceived severity of and perceived susceptibility to a danger would influence their subsequent behavior change [41].

Nonetheless, empirical studies reported mixed findings regarding the persuasive effect of threat messages. For instance, a number of studies on health behavior reported significant effects of threat messages [42,43]. In particular, being exposed to threat messages makes people feel fear and that fear enhances their perception of severity, which in turn motivates them to take some sort of action. However, mixed findings emerged as other studies found no significant relationship between threat message and behavior change [44,45]. Specifically, threat messages may elicit a defensive process when they generate an excessive amount of fear. In this way, people might avoid information about the threat and exhibit maladaptive behaviors. Given the unclear relationship between threat messages and behavioral intention, we propose to explore it in the context of haze mitigation and postulate the following research question:

RQ1: How is attention to haze-related threat messages associated with pro-environmental behavioral intention to engage in haze mitigation?

### 2.4. Interaction of Personal and Media Factors

In addition to the above hypotheses regarding the main effects of TPB concepts and media attention, this study proposes to examine their moderation effects on behavioral intention. The literature on behavioral change suggests that the effectiveness of media messages varies among people with different attributes. For instance, a recent study on public engagement with climate change reported that high efficacy messages were less effective for viewers with strong ecocentrism worldview [18]. In addition, another study on health behavior reported that self-affirmation could moderate the impact of variables in the EPPM on behavioral intention [17]. These studies suggest that individuals’ attributes might serve as moderators in the relationships between media attention and behavioral intention. As such, we expect that the TPB concepts—existing attitude toward behavior, subjective norm, and perceived behavioral control—might have moderating effects on the relationships between individuals’ attention to efficacy/threat messages and their behavioral intention.

Despite a significant number of studies examining efficacy and threat messages separately, the persuasion literature points out that either efficacy or threat messages alone are not enough to elicit behavioral change. Accordingly, various combinations of efficacy and threat messages are created to identify the most effective one. In particular, the EPPM literature indicates that people who are exposed to messages with both high efficacy and high threat content have the highest behavioral intention [11,14]. However, whether such an effect varies among different groups of people remains largely unknown. Thus, this study proposes to examine how the interaction effects between attention to efficacy and threat messages on behavioral intention vary among people with different attitude, social norm, and perceived behavioral control. Correspondingly, three-way interactions among attention to efficacy messages, attention to threat messages and TPB concepts, are also assessed in this study. Due to the dearth of research on the interactions between attention to threat/efficacy messages and TPB concepts, we propose the following research questions:

RQ2: How does the attitude moderate the relationships between attention to haze-related efficacy/threat messages and pro-environmental behavioral intention to engage in haze mitigation?

RQ3: How does the subjective norm moderate the relationships between attention to haze-related efficacy/threat messages and pro-environmental behavioral intention to engage in haze mitigation?

RQ4: How does the perceived behavioral control moderate the relationships between attention to haze-related efficacy/threat messages and pro-environmental behavioral intention to engage in haze mitigation?

## 3. Methods

### 3.1. Data Collection

This research conducted an online survey to collect data during March 2018 in China. As a preliminary study, we employed the snowball-sampling technique to recruit respondents. Specifically, a URL link to the online questionnaire was sent to respondents through instant messages (e.g., QQ, WeChat). Potential respondents were also asked to share the link to their QQ or WeChat contacts. The researchers made efforts to collect nationwide data. A total 432 adults completed questionnaires in this study. Two reversed questions were included in the questionnaire to filter out the invalid respondents. After excluding the invalid questionnaire, we obtained a valid sample of 401 respondents.

### 3.2. Sample

Of the 401 respondents, 62.34% of the respondents were female. The mean age was 27.76 years old, with a range from 18 to 67 years old. Approximately 75.4% had received a college degree. The median monthly household income was 7000 to 9000 CNY.

### 3.3. Measures

***Control variables.*** Demographic variables were used as control variables in this study, including age, gender, education, and monthly household income. 

***Attention to media messages about haze.*** Individuals’ attention to media messages about haze was measured using items adopted from previous studies [20,46]. Attention to efficacy and threat messages about haze was measured respectively. Specifically, *attention to haze-related efficacy messages* was measured by asking respondents to indicate how much attention (1 = *no attention at all*, 7 = very close attention) they paid to pro-environmental messages related to haze in four media channels: newspaper (including print and digital edition), television, the Internet, and social media (M = 4.95, SD = 1.01, Cronbach’s α = 0.65). *Attention to haze-related threat messages* was measured by asking respondent the above statements while replacing pro-environmental messages with environmental crisis messages related to haze (M = 4.92, SD = 1.05, Cronbach’s α = 0.71).

***Attitude toward pro-environmental behavior.*** To measure the attitude toward pro-environmental behaviors, six items were adapted from prior research [47]. Respondents were asked to indicate their agreement with the following statements on a scale from 1 (strongly disagree) to 7 (strongly agree): “I think that engaging in pro-environmental behavior is (a) enjoyable, (b) beneficial, (c) important, (d) worthwhile, (e) easy, (f) compatible with my lifestyle, and (g) satisfying”. A higher average score indicates a more positive attitude toward pro-environmental behavior. (M = 5.65, SD = 1.01, Cronbach’s α = 0.87).

***Social norms.*** The measurement for social norms was adapted from a study by Park and Smith [48]. Respondents were asked to indicate their agreement with statements that their family, close friends, and the general public expect them to engage in pro-environmental behaviors to alleviate haze (1 = *strongly disagree*, 7 = *strongly agree*; M = 5.31, SD = 1.17, Cronbach’s α = 0.87).

***Perceived behavioral control.*** Four items used to measure perceived behavioral control were adapted from a study by Snyder and Rouse [49]. On a scale from 1 (strongly disagree) to 7 (strongly agree), respondents indicated their agreement with the following statements: (a) “I am confident that I can participate in haze mitigation,” (b) “I can control my involvement in haze mitigation,” (c) “I am fully capable of mitigate haze pollution,” and (d) “I am good at leading a green lifestyle” (M = 5.29, SD = 1.05, Cronbach’s α = 0.82).

***Pro-environmental behavioral intention.*** Six items were adopted from prior studies to measure individuals’ pro-environmental behavioral intention to engage in haze mitigation [50,51]. Respondents reported on a 7-point scale (1 = strongly disagree, 7 = strongly agree) their level of agreement with the following statements of behavioral intention in the next six months: (a) “When possible I would ride a bicycle or take public transportation to work or school,” (b) “I intend to buy products in refillable packages,” (c) “When cooking I would use a lid to cover the pot or pan to avoid wasting energy,” (d) “I would recycle used paper and plastic,” (e) “I intend to use less air-conditioning,” and (f) “I would try to convince others of the importance of environmental protection” (M = 5.38, SD = 0.99, Cronbach’s α = 0.78).

### 3.4. Data Analysis

Hierarchical ordinary least squares (OLS) regression analysis was used to test the hypotheses in this study. Demographic variables, including age, gender, education, and household income, were put into the first block. The TPB variables, including attitude, social norms, and perceived behavioral control were entered into the second block. Attention to haze-related efficacy and threat messages was entered into the third block as communication variables.

To examine the moderation effects, the interaction terms were created by multiplying the centered values of the respective main effect variables to reduce potential multicollinearity problems between the interaction term and its components [52]. Then, the hypothesized two-way interaction terms were put into the forth block, including attention to efficacy messages x TPB variables and attention to threat messages x TPB variables. Finally, the hypothesized three-way interaction terms of attention to efficacy messages x attention to threat messages x TPB variables were entered into the fifth block.

## 4. Results

### 4.1. Personal Factors Related to Pro-Environmental Behavioral Intention

Table 1 describes the correlation coefficients among all variables. In this study, Harman’s single-factor test was used to detect the threat of common method bias (CMB). In the analysis, all items (measuring latent variables) were loaded into one common factor. The results showed that the total variance for a single factor is 33.236%, which is less than 50%, suggesting that CMB did not affect our data. Table 2 shows the results from the OLS regression analysis. The results in this study are based on the final model (Model 5) in the hierarchical ordinary least squares (OLS) regression analysis. Regarding the hypothesized relationships between TPB variables and the pro-environmental behavioral intention, the results revealed that attitude (*β* = 0.29, *p* < 0.001), social norms (*β* = 0.22, *p* < 0.001), and perceived behavioral control (*β* = 0.25, *p* < 0.001) were all positively related to pro-environmental behavioral intention. Hence, H1, H2, and H3 were all supported.

### 4.2. Media Factors Related to Pro-environmental Behavioral Intention

In terms of the hypothesized relationships between media attention and pro-environmental behavioral intention, the results showed that attention to positive media messages about haze was positively related to pro-environmental behavioral intention (*β* = 0.15, *p* < 0.01), which supported H4. Attention to negative media messages about haze had no significant relationship with behavioral intention, which answered RQ1.

### 4.3. Interaction Effects

**Figure 2:**
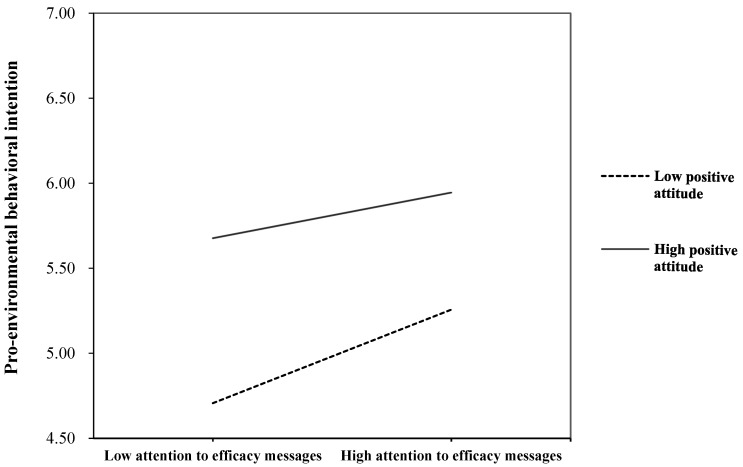
Pro-environmental behavior intention by attitude and attention to efficacy messages.

Regarding the moderating effects, results revealed that attitude could moderate the relationship between attention to efficacy messages and behavioral intention (*β* = −0.19, *p* < 0.05), which answered RQ2. As shown in Figure 2, increasing attention to efficacy messages would lessen the behavioral intention gaps among people with different levels of positive attitude toward performing behaviors.

**Figure 3:**
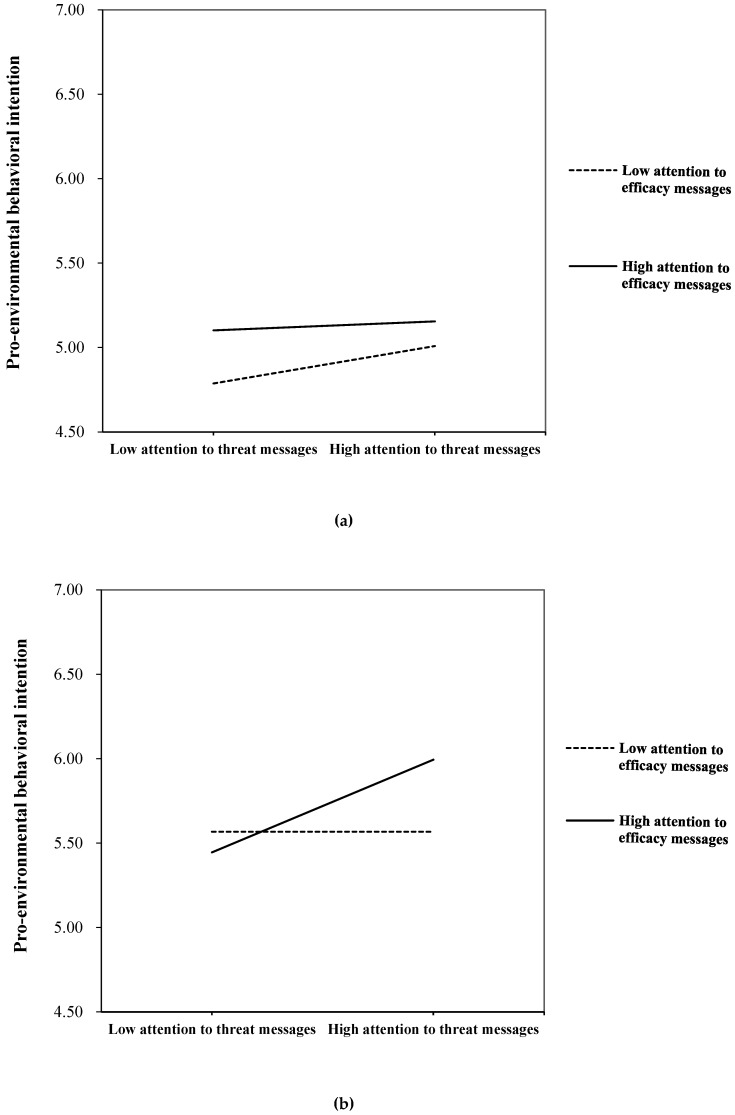
(**a**) Pro-environmental behavior intention by attention to efficacy messages and threat messages, when social norms are low. (**b**) Pro-environmental behavior intention by attention to efficacy messages and threat messages, when social norms are high.

In terms of the moderation effects of subjective norms, the results showed that there was no significant two-way interaction with attention to either efficacy messages or threat messages. But the three-way interaction among attention to efficacy messages, attention to threat messages, and subjective norm on behavioral intention to engage in haze mitigation was significant (*β* = 0.16, *p* < 0.05), which answered RQ3. This positive three-way interaction suggested that the relationship between media attention and behavioral intention varies across different levels of subjective norms. The three-way interactive relationship of subjective norms was plotted in Figure 3a,b.

As shown in the figures, in explaining one’s behavioral intention to engage in haze mitigation, the interplay between attention to efficacy and threat messages about haze marked distinctive patterns when people have different levels of subjective norms. Figure 3a showed that among people who have less subjective norms, increasing attention to threat messages about haze effects would decrease behavioral intention gaps among people who pay different amounts of attention to efficacy messages. In particular, among people who have less subjective norms and pay less attention to efficacy messages, increasing attention to threat messages would enhance their behavioral intention greatly (see bottom line in Figure 3a). However, Figure 3b revealed an opposite pattern for the respondents who have more subjective norms. Specifically, among people who had more subjective norms, increasing attention to threat messages about haze effects would magnify behavioral intention gaps among people who paid different amounts of attention to efficacy messages. For those who had more subjective norms and paid more attention to efficacy messages, increasing attention to threat messages would enhance their behavioral intention greatly (see upper line in Figure 3b). For those who had more subjective norms but paid less attention to efficacy messages, increasing attention to negative messages about haze would not change their behavioral intention significantly (see bottom line in Figure 3b).

**Figure 4:**
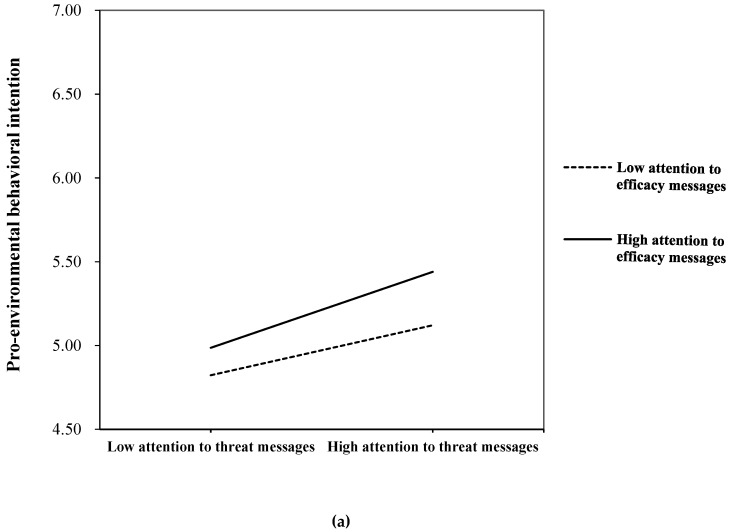

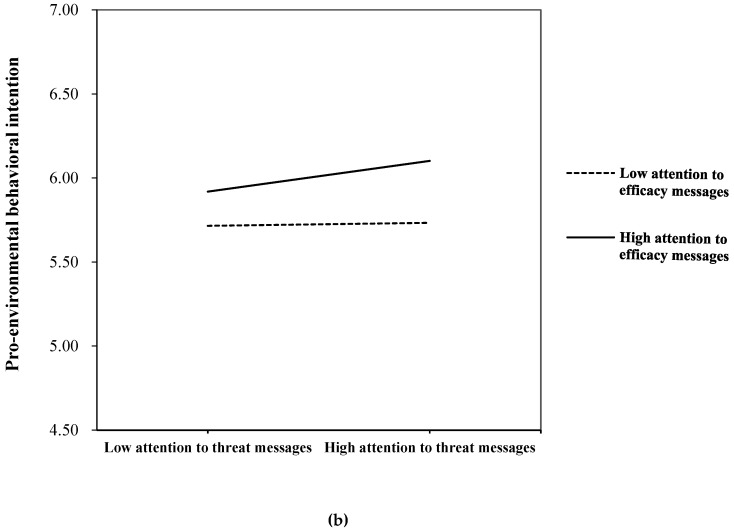
(**a**) Pro-environmental behavior intention by attention to efficacy messages and threat messages, when perceived behavioral control is low. (**b**) Pro-environmental behavior intention by attention to efficacy messages and threat messages, when perceived behavioral control is high.

RQ4 considers the moderation effects of perceived behavioral control. The results showed that there was no significant two-way interaction between perceived behavioral control and media attention. But the three-way interaction among attention to efficacy and threat messages, and perceived behavioral control on behavioral intention was significant (*β* = −0.23, *p* < 0.01), which answered RQ4. The three-way interactive relationship of PBC was plotted in Figure 4a,b.

In Figure 4a, among people with less perceived behavioral control to perform pro-environmental behavior, increasing attention to threat messages about haze effects would magnify behavioral intention gaps among people who paid different amounts of attention to efficacy messages. In particular, among people who had less perceived behavioral control but paid more attention to efficacy messages, increasing attention to threat messages would enhance their behavioral intention greatly (see upper line in Figure 4a). Figure 4b showed a similar pattern for people with more perceived behavioral control. However, for those who had more perceived behavioral control but paid less attention to efficacy messages, increasing attention to threat messages about haze would not change their behavioral intention greatly (see bottom line in Figure 4b).

## 5. Discussion

Findings from this study supported the TPB variables in predicting behavioral intention in the context of haze mitigation. Consistent with previous studies [3,8], attitude, subjective norm, and perceived behavioral control positively predicted pro-environmental behavioral intention. These results reveal that people are more likely to engage in haze mitigation if they perceive the behavior to be important and beneficial. The positive association between subjective norm and behavioral intention suggests that people tend to engage in haze mitigation if they believe that others expect them to do so. Moreover, the nature of collective action in addressing environmental issues may explain the significant relationship between subjective norm and intention to engage in haze mitigation, as people tend to feel more social pressure to take collective action [53]. We speculate that individuals’ perception of behavioral control may reflect the increasing availability of public transportation in China, as taking public transportation is considered one of the most feasible solution to alleviate haze pollution.

As aforementioned above, this study extended the TPB by examining the effects of media content instead of communication channels. Our analyses yield mixed results for the relationships between attention to different media messages and pro-environmental behavioral intention. Specifically, attention to haze-related efficacy messages was positively associated with pro-environmental behavioral intention. This finding suggests that efficacy information about haze mitigation can be important for promoting individuals’ participation. Since prior studies have demonstrated the positive relationship between individuals’ efficacy and behavior [54,55], it is unsurprising that attention to haze-related efficacy messages predicts behavioral intention to engage in haze mitigation.

However, we found that attention to haze-related threat messages was not related to haze mitigation. This finding comports with prior studies that threat messages may not elicit behavioral change [11,56]. Theoretical literature on message design points out when fear appeals trigger a high perceived threat alongside a low perceived efficacy, individuals would subsequently engage in defensive avoidance [13]. Besides, the characteristics of haze issue might explain the non-significant association between attention to threat messages and behavioral intention. Individuals might develop fatalistic beliefs toward fighting haze when they are exposed to excessive threat information, as haze is addressed as a personal risk that poses direct threats to humans.

Moreover, findings from this study revealed that increasing attention to efficacy messages would narrow the behavioral intention gaps between people with different attitudes toward pro-environmental behaviors. In particular, attention to haze-related efficacy messages had positive effects on individuals’ pro-environmental behavioral intention, irrespective of how much positive attitude they had. The positive effects are notably stronger among people who have less positive attitude, as compared to those who have more positive attitude. It is possible that people with more positive attitude have stronger behavioral intention than those with less positive attitude, even if they pay no attention to efficacy messages. Thus, increasing attention might not enhance their behavioral intention significantly, due to their already higher intention. This implies that haze-related efficacy messages could be much more beneficial for people with less positive attitude toward performing pro-environmental behavior.

The interaction between attention to haze-related messages and subjective norms on pro-environmental behavioral intention reveals a variation of media effects among people with different levels of subjective norms. For people who feel less social influence from others, attention to haze-related threat messages had stronger effects on pro-environmental behavioral intention when they pay less attention to efficacy messages. One possible explanation may lie in individual differences in perceived social influences. Prior studies demonstrated that individualistic people hold less stringent social norms and focus more on self-interest, while collectivistic people hold more social norms and value collective-interest more [57,58]. In other words, people with less subjective norms have more individualistic value orientation, whereas people with more subjective norms have more collectivistic value orientation. Driven by the pursuit of self-interest, individualistic people tend to be more sensitive about threat to personal safety. Compared to individualistic people who pay more attention to efficacy messages, those who pay less attention to efficacy messages would feel more uncertainty and anxious about the risks related to haze pollution. Thus, it is unsurprising that people with less subjective norms would be significantly influenced by threat information especially when they lack efficacy information.

Comparatively, the results show that for people who feel more social influence from others, the stronger effects of attention to haze-related threat messages on behavioral intention occur when they pay more attention to efficacy messages. The individual difference in perceived social influence accounts for this finding as well. As aforementioned, people with more subjective norms value collective interest more. For those people who have more subjective norms, an immersion in threat information would make them feel more about the urgency of taking action, as they are concerned that both themselves and others in the whole society will be negatively affected by haze pollution. In this case, paying attention to efficacy messages helps to a great extent, as they can learn about how to take action.

Our study also found that the relationship between media attention and behavioral intention varies among people with different levels of perceived behavioral control. For people with less perceived behavior control over haze mitigation, attention to haze-related threat messages had positive effects on individuals’ pro-environmental behavioral intention, regardless of how much attention that they pay to efficacy messages. As compared to those who pay less attention to efficacy messages, the positive effects were much stronger among people who pay more attention to efficacy messages. A similar pattern was found among people with more perceived behavioral control as well. These findings suggest that when people pay a lot of attention to both efficacy and threat messages, their intention to engage in haze mitigation would be very high, irrespective of the levels of their perceived behavioral control. This is consistent with prior studies on behavioral change, which demonstrated the significant effects of messages containing both high efficacy and high threat [11,59].

## 6. Theoretical and Practical Implications

Our study has several theoretical contributions. First, this study extends the TPB by taking communication variables into account. Unlike other TPB extension studies, we shift the focus of inquiry from examining the effects of attention to different media platforms to assessing the effects of attention to different media messages on behavior. In particular, findings from our study develop the TPB by indicating that attention to different media messages can offer different effects on behavioral intention. Second, previous studies on efficacy and threat messages mostly focus on the effects of their various combinations on behavior, whereas very few of them have examined how the various combinations of efficacy and threat messages work among people with different characteristics. This study is among the first to examine how the interaction effects between attention to efficacy and threat messages vary among different groups of people. Specifically, the TPB variables—attitude, subjective norms, and perceived behavioral control—were included as the moderators. Findings from this study reveal that the relationships between paying attention to haze-related efficacy/threat messages and behavioral intention vary among people with different attitude, subjective norms, and perceived behavioral control toward haze mitigation. By examining the moderating effects of the TPB variables, this study contributes to the theoretical literature on the TPB. Besides, a number of recent studies have used the TPB to examine the determinants of pro-environmental behavioral intention to save water [60], to engage in green buying [8], and to fight against climate change [61]. The current study extends the application of the TPB on haze mitigation, a serious environmental issue in China. As such, this study has made a significant contribution to the body of knowledge on the application of the TPB in the context of environmental issues.

In terms of practical contributions, our findings provide several specific suggestions for environmental campaign management. Policymakers and practitioners might achieve their goals more effectively by tailoring media messages containing different combinations of efficacy and threat information to promote pro-environmental behavioral intention among the public. For people with a less positive attitude, providing efficacy messages on how to alleviate haze would greatly increase their behavioral intention. For people with less normative beliefs toward haze mitigation, an offer of threat messages would be more effective in increasing their behavioral intention than an offer of efficacy messages. In contrast, an offer of haze-related efficacy messages rather than threat messages would be more effective in promoting behavioral intention for people with more subjective norms. Besides, providing both efficacy and threat messages would greatly enhance the behavioral intention for people who have less perceived behavioral control toward haze mitigation.

## 7. Limitations and Future Research Directions

This study has its limitations. First, our analysis of cross-sectional data prevents causal inference. Future studies are suggested to conduct a longitudinal survey or experimental design to clarify causation. Specifically, an experimental design of efficacy and threat messages would be more valid in interpreting the effects of media messages. Second, this study utilized snowball-sampling to recruit respondents. Further research is necessary by utilizing a representative sample. Besides, our study examines pro-environmental behavioral intention as a one-dimension construct. Future studies could replicate the present study by making a comprehensive examination of pro-environmental behavioral intention in private-sphere and public-sphere respectively.

In addition, future works could consider building on our work by testing the moderating effects of the TPB in the domain of environmental or non-environmental contexts such as health-related issues. An understanding of how people with different levels of attitude toward health behaviors, social norms, and perceived behavioral control react to health campaigns would be instructive for the implementation of health promotion programs.

## 8. Conclusions

Overall, this study contributed to existing studies by integrating the communication variables with the TPB variables that might motivate public engagement in haze mitigation. Unlike other TPB extension studies underlining the distinction of different media platforms, this study highlighted the effects of media content. Thus, rather than examining media attention across different platforms, this study operationalized media attention in terms of the amount of attention that people paid to haze-related efficacy and threat messages. Results indicated that attitude, subjective norm, perceived behavioral control, and attention to haze-related efficacy messages were positively related to individuals’ behavioral intention to engage in haze mitigation. Moreover, attitude moderated the influence of efficacy messages on behavioral intention. The interaction effects of attention to efficacy and threat messages on behavioral intention were significantly moderated by subjective norms and perceived behavioral control.

## Figures and Tables

**Table 1:** Bivariate correlation of all variables.

	1	2	3	4	5	6	7	8	9
1. Age	----								
2. Gender	−0.07								
3. Education	−0.19 **	−0.08							
4. Income	0.12 *	−0.17 **	0.18 ***						
5. Attitude	0.17 **	0.14 **	−0.03	−0.003					
6. Social norms	0.16 **	−0.04	0.01	0.06	0.47 ***				
7. Perceived behavioral control	0.15 **	0.04	0.03	0.05	0.61 ***	0.68 ***			
8. Attention to efficacy messages	0.08	−0.01	−0.02	0.05	0.29 ***	−0.28 ***	0.27 ***		
9. Attention to threat messages	0.03	0.01	−0.07	0.12 *	0.34 ***	0.37 ***	0.40 ***	0.70 ***	
10. Pro-environmental behavioral intention	0.16 **	0.07	0.001	−0.002	0.60 ***	0.54 ***	0.59 ***	0.32 ***	0.32 ***

Note: * *p* < 0.05, ** *p* < 0.01, *** *p* < 0.001.

**Table 2:** Hierarchical regression analysis predicting pro-environmental behavioral intention.

	Zero-order	Model 1	Model 2	Model 3	Model 4	Model 5
Block 1: Demographic variables						
Age	0.16 **	0.18 ***	0.04	0.05	0.05	0.04
Gender	0.07	0.08	0.02	0.03	0.04	0.04
Education	0.001	0.05	0.02	0.02	0.01	0.01
Income	−0.002	−0.03	−0.03	−0.03	−0.03	−0.03
Incremental R (%)		3.50 ***				
Block 2: TPB variables						
Attitude	0.60 ***		0.36 ***	0.34 ***	0.29 ***	0.27 ***
Social norms	0.54 ***		0.22 ***	0.21 ***	0.22 ***	0.19 **
Perceived behavioral control	0.60 ***		0.22 ***	0.22 ***	0.25 ***	0.32 ***
Incremental R (%)			43.80 ***			
Block 3: Communication variables						
Attention to efficacy messages	0.32 ***			0.15 **	0.15 **	0.16 **
Attention to threat messages	0.32 ***			−0.07	−0.03	−0.03
Incremental R (%)				1.30 ***		
Block 4: Two-way interactions						
Efficacy messages *threat messages attention					0.07	0.05
Efficacy messages attention* ATT					−0.15 *	−0.19 *
Threat messages attention* ATT					0.04	0.09
Efficacy messages attention* SN					0.00	0.09
Threat messages attention* SN					0.03	0.06
Efficacy messages attention* PBC					0.08	0.02
Threat messages attention* PBC					0.07	−0.001
ATT * SN					−0.07	−0.13
ATT * PBC					0.004	0.04
PBC * SN					0.04	0.09
Incremental R (%)					2.30 ***	
Block 5: Three-way interactions						
Efficacy messages * Threat messages * ATT						0.06
Efficacy messages * Threat messages * SN						0.16 *
Efficacy messages * Threat messages * PBC						−0.23 **
Incremental R (%)						1.40 ***
Total R (%)						52.30 ***

Note: * *p* < 0.05, ** *p* < 0.01, *** *p* < 0.001.
